# Antiplatelet and Antithrombotic Effects of *Epimedium koreanum* Nakai

**DOI:** 10.1155/2021/7071987

**Published:** 2021-04-16

**Authors:** Muhammad Irfan, Tae-Hyung Kwon, Dong-Ha Lee, Seung-Bok Hong, Jae-Wook Oh, Sung-Dae Kim, Man Hee Rhee

**Affiliations:** ^1^Department of Veterinary Medicine, College of Veterinary Medicine, Kyungpook National University, Daegu 41566, Republic of Korea; ^2^Department of Oral Biology, College of Dentistry, University of Illinois at Chicago, Chicago 60607, IL, USA; ^3^Chuncheon Bio Industry Foundation, Chuncheon 24232, Republic of Korea; ^4^Department of Clinical Laboratory Science, Chungbuk Health & Science University, Chungbuk 28150, Republic of Korea; ^5^Department of Biomedical Laboratory Science: and Molecular Diagnostics Research Institute, Namseoul University, Cheonan 31020, Republic of Korea; ^6^Department of Stem Cell and Regenerative Biotechnology, KIT, Konkuk University, Seoul 05029, Republic of Korea; ^7^Research Center, Dongnam Institute of Radiological and Medical Sciences, Busan 46033, Republic of Korea

## Abstract

*Background and Objective*. *Epimedium koreanum* Nakai is a medicinal plant known for its health beneficial effects on impotence, arrhythmia, oxidation, aging, osteoporosis, and cardiovascular diseases. However, there is no report available that shows its effects on platelet functions. Here, we elucidated antiplatelet and antithrombotic effects of ethyl acetate fraction of *E. koreanum*. *Methodology*. We analyzed the antiplatelet properties using standard *in vitro* and *in vivo* techniques, such as light transmission aggregometry, scanning electron microscopy, intracellular calcium mobilization measurement, dense granule secretion, and flow cytometry to assess integrin *α*_IIb_*β*_3_ activation, clot retraction, and Western blot, on washed platelets. The antithrombotic effects of *E. koreanum* were assessed by arteriovenous- (AV-) shunt model in rats, and its effects on hemostasis were analyzed by tail bleeding assay in mice. *Key Results*. *E. koreanum* inhibited platelet aggregation in agonist-stimulated human and rat washed platelets, and it also reduced calcium mobilization, ATP secretion, and TXB2 formation. Fibrinogen binding, fibronectin adhesion, and clot retraction by attenuated integrin *α*_IIb_*β*_3_-mediated inside-out and outside-in signaling were also decreased. Reduced phosphorylation of extracellular signal-regulated kinases (ERK), Akt, PLC*γ*2, and Src was observed. Moreover, the fraction inhibited thrombosis. HPLC results revealed that the fraction predominantly contained icariin. *Conclusion and Implications*. *E. koreanum* inhibited platelet aggregation and thrombus formation by attenuating calcium mobilization, ATP secretion, TXB2 formation, and integrin *α*_IIb_*β*_3_ activation. Therefore, it may be considered as a potential candidate to treat and prevent platelet-related cardiovascular disorders.

## 1. Introduction

Cardiovascular disease (CVD) is the leading cause of death in modern societies, especially in Western and other developed countries [1]. The World Health Organization stated that CVD accounted for 30% of all deaths in 2005, and in Europe, it remains the primary cause of 42% of mortalities in men and 52% in women [2]. Platelets are considered as major pathological risk factors for CVDs, such as coronary artery disease and atherosclerosis. Pathophysiologic hyper-activation of platelets leads to platelet aggregation and thrombus formation, which ultimately contribute to blood vessel stenosis, ischemia, and myocardial infarction [3]. Several drugs are available to treat and prevent platelet-related cardiovascular ailments; however, their side effects often outweigh their benefits. For example, aspirin causes gastric ulcers and clopidogrel may produce aplastic anemia and thrombocytopenic purpura [4], whereas a significant part of the population is resistant to these most commonly used anti-platelet agents [5].

Using natural products and their bioactive natural compounds, including ethnomedicinal applications for CVD treatment and prevention, has been increased [6, 7]. Similarly, several natural products, including traditional Mediterranean diet and medicinal plants, have also been reported for their cardio-protective and anti-platelet effects in primary and secondary CVD prevention [8–11]. *Epimedium koreanum* Nakai, commonly known as horny goat weed, is native to Korea and China, which has been extensively used as a nutraceutical in functional foods or as phyto-pharmaceutical agent for preventing and curing some serious and fatal illnesses, such as CVD and osteoporosis, and to improve neurological and sexual functions [12–14]. Zhang et al. [15] have prepared and explored the anti-oxidant activity of the several fractions of *E. koreanum* Nakai and found that n-butanol fraction and ethyl acetate fraction (EAF) were having most scavenging activity with maximum content of icariin in EAF.

However, no study has shown its possible anti-platelet mechanism involved in platelet-related cardiovascular disorders. Here, we evaluated the anti-platelet and anti-thrombotic effects of EAF of *E. koreanum* and examined its inhibitory effects on normal platelet functions.

## 2. Materials and Methods

### 2.1. Procurement and Extraction of *E. koreanum* Nakai

*E. koreanum* was purchased from Omniherb of Gyeongsan (Gyeongbuk province, Korea). The samples were pulverized to 80 mesh size using Sung Chang Machine (ACM10-INCH, Namyangju, Korea) and then fractionated, according to the organic solvent's polarity, to measure the physiologic activity. Then, 1 L of methanol was added to 100 g of the ground powder and extracted at 150 rpm for 24 hours using a shaker. After centrifugation at 4°C and 8000 rpm for 10 minutes, and filtration, the aforementioned process was repeated twice to obtain the methanol extract. Distilled water and hexane in a 1 : 1 ratio were added to the methanol extract and processed for 24 hours in a shaker; then sonication, centrifugation, and concentration were repeated twice to obtain the hexane (0.82 g) extract. By repeating the aforementioned process, chloroform (0.79 g), ethyl acetate (0.83 g), butanol (1.55 g), and water (8.61 g) extracts were obtained. The yield was measured by lyophilizing the extract and dissolving in DMSO and used for efficacy verification and then stored at −80 °C until use.

### 2.2. Analysis of Phenolic Compounds Using HPLC

Analysis of the major phenolic compounds in *E. koreanum* ethyl acetate fraction was conducted using HPLC (Alliance, Waters, USA). An Eclipse plus C18 column (Agilent Technologies Ltd.; 4.6 × 250 mm; particle size, 5 *μ*m) was used for analysis of phenolic acid and flavonoid content. The mobile phases were 0.1% formic acid (v/v) in 10% acetonitrile (solvent A) and 0.1% formic acid (v/v) in 90% acetonitrile (solvent B). All reagents used were of HPLC grade. The gradient followed the following order: 0–6 min 0% B, 6–31 min 10%–90% B, 31–41 min 20%–80%, 41–45 min 50%–50% B, and 45–50 min 0% B. The separated phenolic compounds were detected using a photodiode array detector at 280 nm.

### 2.3. Preparation of Washed Human and Rat Platelets

Human platelet-rich plasma (PRP) collected from healthy volunteers, who provided informed consent, was obtained from the Korean Red Cross Blood Center (KRBC, Changwon, Korea), and its experimental use was approved by KRBC and the Korea National Institute for Bioethics Policy Public Institutional Review Board (PIRB17-1019-03). The washed human and rat platelets were prepared as previously described [16].

### 2.4. Platelet Aggregation Assay and Scanning Electron Microscope (SEM) Analyses

The extent of platelet aggregation was assessed by following the standard procedure of light transmission aggregometry using a Chrono-log aggregometer (Havertown, PA, USA), as previously described [17]. Briefly, the washed platelets (3 × 10^8^ cells/mL) were pre-incubated with vehicle (DMSO, maintained at <0.1%) or different concentrations of EAF for 1 min in the presence of 1 mM calcium chloride (CaCl_2_), followed by stimulation with various agonists (collagen, ADP, or thrombin) for 5 min with continuous stirring at 37°C.

A field emission SEM was used to assess platelet shape change and aggregation by obtaining ultrastructure images as previously described [17,18].

### 2.5. Arteriovenous Shunt Model

The anti-thrombotic activity of the *E. koreanum* fraction was assessed in a rat extracorporeal shunt model as previously described [9,17]. Briefly, rats were orally administered with the saline (control), EAF (100–300 mg/kg), or ASA (50 mg/kg) once daily for 3 days. Then, 2 h after the last administration, rats were anesthetized and a shunt was placed for 15 min after initiating extracorporeal circulation. Subsequently, blood flow was stopped and the formed thrombus was weighed.

### 2.6. In Vivo Bleeding Assay

Male mice were divided into three treatment groups (*n* = 5) and intraperitoneally administered with saline (control), ASA (50 mg/kg), or EAF (300 mg/kg) once daily for 3 days. One hour after the last administration, mice were anesthetized, and tail bleeding assay was performed as previously described [19].

### 2.7. Statistical Analysis

Data were analyzed by one-way analysis of variance, followed by measuring statistically significant differences using Dunnett's post hoc test (SAS Institute Inc., Cary, NC, USA). All data were presented as the mean ± standard deviation (SD). A *p* value of ≤0.05 was considered statistically significant.

## 3. Results

### 3.1. *E. koreanum* Inhibits Agonist-Stimulated Platelet Aggregation

**Figure 1:**
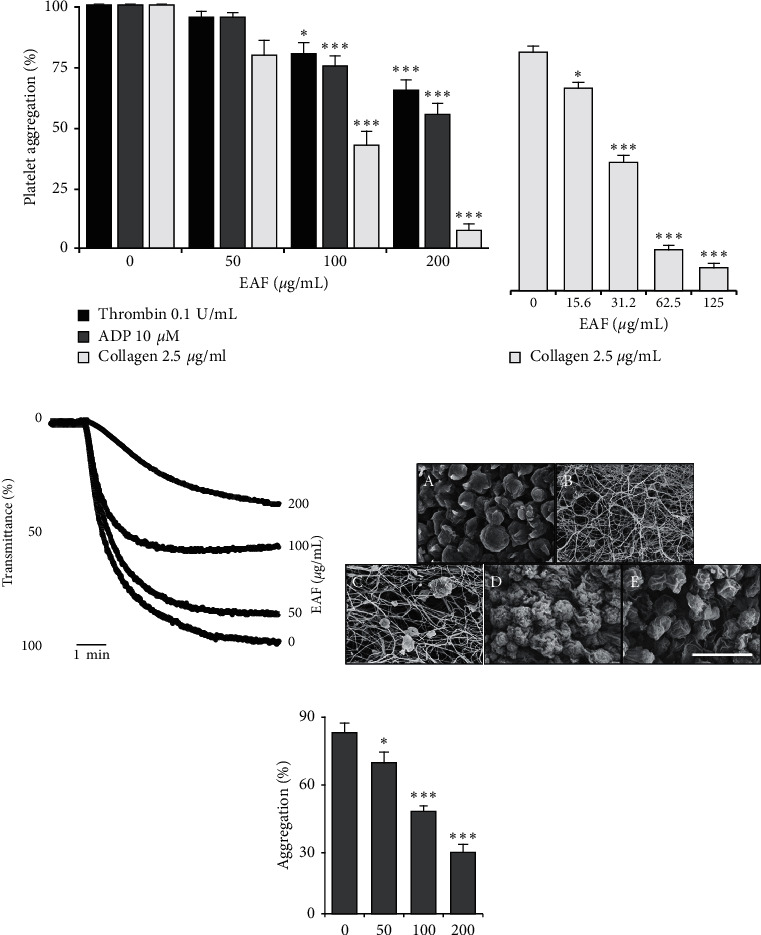
*E. koreanum* inhibits agonist-induced human and rat platelet aggregation. Collagen-, ADP-, or thrombin-stimulated washed platelets ((a) and (d) from rats (3 × 10^8^ cells/mL); (b) from humans (5 × 10^8^ cells/mL)) pretreated with vehicle or various concentrations of *E. koreanum* ethyl acetate fraction (EAF) in the presence of 1 mM CaCl_2_. (c, e) Rat PRP (5 × 10^8^ cells/mL) was incubated with vehicle or various concentrations of extract in the presence of 10-mM CaCl_2_ for 1 min and then stimulated with ADP (25 *μ*M) for 5 min. (d) Representative SEM images (5000×) of collagen (2.5 *μ*g/mL)-stimulated rat platelets pre-treated with vehicle or various concentrations of EAF ((A) resting, (B) vehicle, (C) 50 *μ*g/mL, (D) 100 *μ*g/mL, and (E) 200 *μ*g/mL). The scale bar represents 5 µm. The graphs show mean ± SD values from at least four independent experiments. *∗p* < 0.05 and *∗∗∗p* < 0.001 versus control.

The fraction was tested against several agonists, that is, thrombin-, ADP-, and collagen-stimulated rat platelet aggregation, and significant and dose-dependent inhibitory effects were found compared with vehicle-treated (control) platelets, whereas it has more effectively inhibited collagen-stimulated platelet aggregation (Figure 1(a)). A similar trend has been also seen in collagen-stimulated human platelet aggregation with significant and dose-dependent inhibition, in which the fraction was more potent compared with rat platelets (Figure 1(b)). The fraction also potently inhibited ADP-induced aggregation measured in rat PRP, in a dose-dependent manner (Figure 1(c) and 1(e)). Fraction-treated rat platelets were also analyzed under a SEM, which showed the clear dose-dependent inhibition of platelet aggregation, with increased *E. koreanum* concentration, whereas the platelets treated with only the vehicle and agonist (collagen) caused full platelet activation and aggregation, leading to fibrin meshwork formation (Figure 1(d)).

### 3.2. *E. koreanum* Reduced Calcium Mobilization, Dense Granule Secretion, and Thromboxane-B2 Production

**Figure 2:**
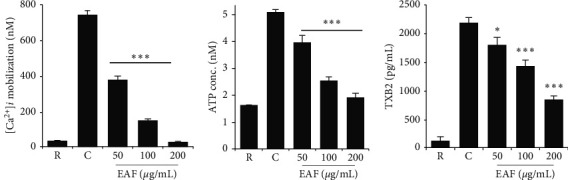
*E. koreanum* inhibits (Ca^2+^)_i_ mobilization and reduces ATP release and TxB2 production. (a) Fura 2/AM-loaded rat platelets (3 × 10^8^ cells/mL) pre-treated with vehicle or different concentration of EAF and stimulated with collagen (2.5 *μ*g/mL) for 3 min. Assessment of ATP concentration (b) and thromboxane-B2 production (c) was done in a supernatant of stimulated washed platelets (3 × 10^8^ cells/mL) suspension pre-treated with vehicle or various EAF concentrations and stimulated with collagen for 5 min on a luminometer or TxB2 ELISA kit, respectively. Results are represented as mean ± SD values from at least four independent experiments. *∗p* < 0.05 and *∗∗∗p* < 0.001 versus control. R: resting; C: control.

Intracellular calcium mobilization ([Ca^2+^]_*i*_) was observed to be decreased in a significant and dose-dependent manner in *E. koreanum*-pre-treated platelets, thereby inhibiting platelet activation (Figure 2(a)). It also dose-dependently reduced ATP secretion and thromboxane-B2 release compared with vehicle-treated rat platelets (Figures 2(b) and 2(c)).

### 3.3. Inhibitory Effects of *E. koreanum* on Fibrinogen Binding, Fibronectin Adhesion, and Clot Retraction Kinetics

**Figure 3:**
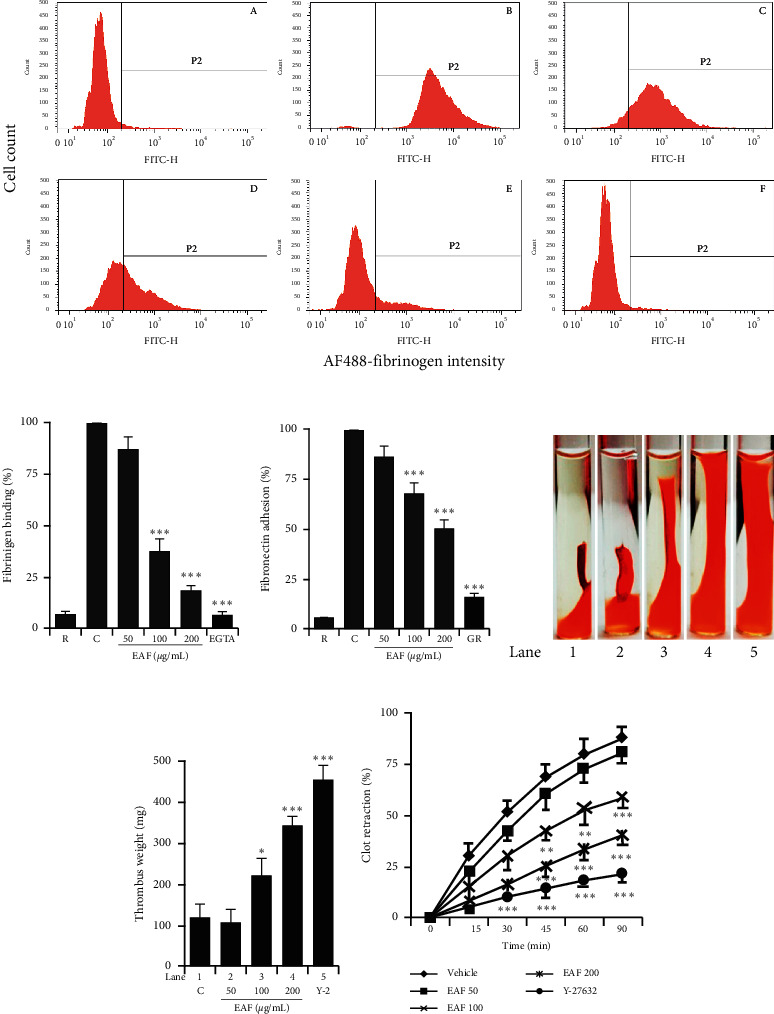
*E. koreanum* inhibits integrin *α*_IIb_*β*_3_-mediated inside-out and outside-in signaling. ((a) and (b)) Flow cytometric measurements of fibrinogen binding in rat platelets (3 × 10^8^ cells/mL) treated with vehicle or different concentrations of *E. koreanum* fraction (EAF), or EGTA ((A) resting, (B) vehicle, (C) 50 *μ*g/mL, (D) 100 *μ*g/mL, (E) 200 *μ*g/mL, and (F) 10 *μ*M EGTA)), and stimulated with collagen (b–f). (c) Results of fibronectin adhesion assay, which was performed using an assay kit according to the manufacturer's instructions and by following the procedure described in the methods section. (d) *In vitro* effect of *E. koreanum* on clot retraction for 2 h at room temperature after thrombin addition and photographed at 15 min intervals. Representative images of clot retraction at 90 min after thrombin addition with or without EAF. Y-27632 (ROCK inhibitor) was used as a control. Lanes 1–5 correspond to lanes 1–5 in (e). (f) Clot retraction kinetics were measured by ImageJ software, and the clot surface areas were plotted as a percentage of retraction. Bar graphs summarizing the inhibitory effect of EAF on fibrinogen binding to integrin *α*_IIb_*β*_3_ (B), fibronectin adhesion (C), clot retraction (E), and kinetics of clot retraction (F). Results are shown as mean ± SD values from at least four independent experiments. *∗p* < 0.05,^*∗∗*^*p* < 0.01, and *∗∗∗p* < 0.001 versus control. R: resting; C: control; GR: GR155053; Y-2: Y-27632.

Taking further insight to its anti-platelet mechanism, we evaluated the effects of the fraction on integrin *α*_IIb_*β*_3_ activation. We found that *E. koreanum* effectively inhibited fibrinogen binding to integrin *α*_IIb_*β*_3_, compared with the washed vehicle-treated rat platelets (Figures 3(a) and 3(b)). Similarly, the fraction significantly inhibited platelet spreading on adhesive ligands, that is, fibronectin (Figure 3(c)). Next, we analyzed the inhibitory effects of the fraction on thrombin-stimulated clot retraction in rat PRP. Our results showed that *E. koreanum* dose-dependently and time-dependently inhibited clot retraction compared with the vehicle-treated control (Figures 3(d)–3(f)).

### 3.4. The Effects of *E. koreanum* on Phosphorylation of Src, MAPK, Akt, and PLC*γ*2

**Figure 4:**
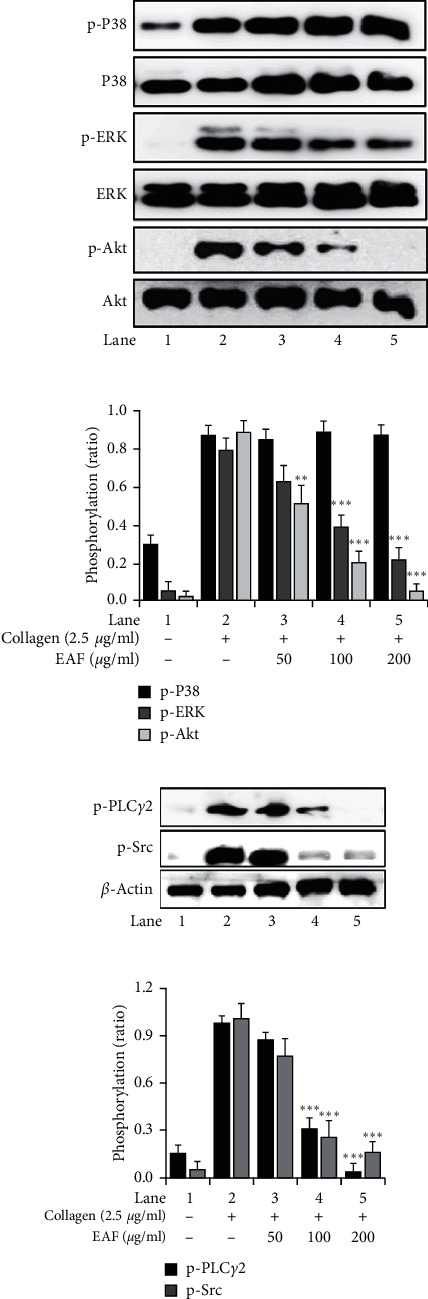
*E. koreanum* attenuates phosphorylation levels of ERK, Akt (a), Src, and PLC*γ*2 (b). Immunoblotting was conducted to analyze the phosphorylation of signaling molecules extracted from the lysates of collagen-stimulated washed rat platelets (3 × 10^8^ cells/mL) that were pretreated with vehicle or *E. koreanum* fraction (EAF). Representative immunoblot images and data (mean ± SD) from at least four independent experiments are shown. Lanes 1–5 of blot images correspond to each bar graph, respectively. *∗p* < 0.05 and *∗∗∗p* < 0.001 versus the agonist-treated group.

Further mechanistic aspects were explored by assessing the phosphorylation of key molecules involved in platelet aggregation. The mitogen-activated protein kinase (MAPK) pathway was analyzed, especially ERK and P38^MAPK^, along with several other molecules like Src, PLC*γ*2, and Akt. The results significantly reduced ERK, Src, PLC*γ*2, and Akt phosphorylation but not P38^MAPK^ in washed rat platelets, showing the potential inhibitory mechanism of *E. koreanum* on platelet activation (Figures 4(a) and 4(b)).

### 3.5. *E. koreanum* Inhibits Thrombus Formation and Modulates Hemostasis

**Figure 5:**
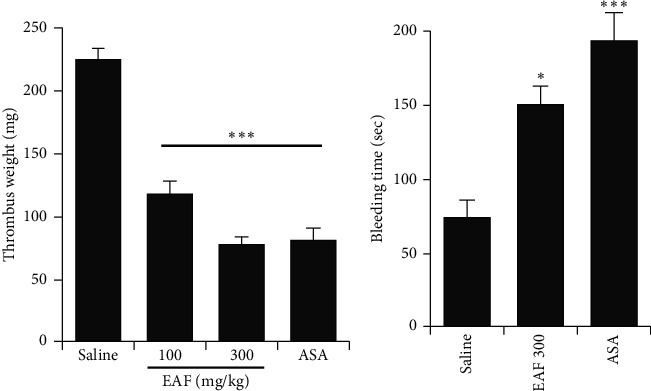
*E. koreanum* inhibits thrombus formation and modulates hemostasis. (a) *In vivo* evaluation of anti-thrombotic activity and determination of thrombus weight in AV-shunt model of rats that were orally administered with saline, ethyl acetate fraction of *E. koreanum* (EAF; 100–300 mg/kg), or ASA (50 mg/kg). (b) Results of tail bleeding assay for homeostasis measurement in mice administered with EAF (300 mg/kg), ASA (50 mg/kg), or saline (*n* = 5 in each group). Graph shows mean ± SD values from at least five independent experiments performed. *∗p* < 0.05 and *∗∗∗p* < 0.001 versus control.

The AV-shunt model results revealed that *E. koreanum* effectively reduced thrombus weight in rats in a significant and dose-dependent manner, as compared with the control group. Similar inhibition was observed in ASA-treated rats (Figure 5(a)). Bleeding time was also increased in *E. koreanum*-treated and ASA-treated mice, as compared with the control group (Figure 5(b)). These results indicate that the fraction attenuated platelet activation and inhibited thrombus formation.

### 3.6. Constituents of *E. koreanum* Ethyl Acetate Fraction

**Figure 6:**
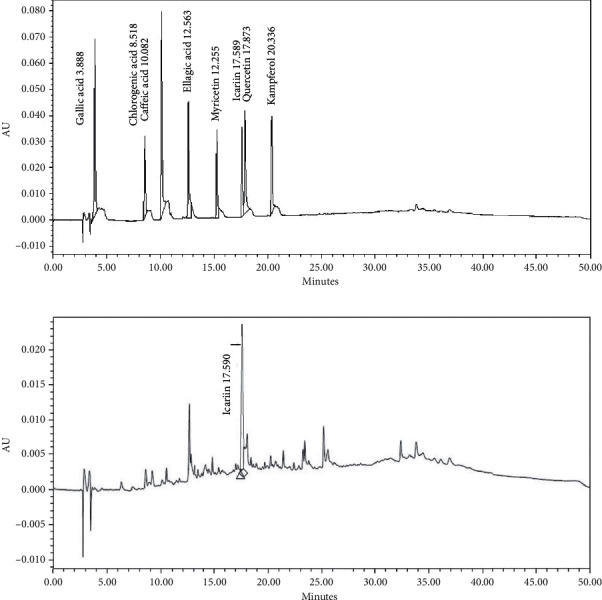
Chemical constituents of *E. koreanum* ethyl acetate fraction. (a) Chromatograms of the standard solution. (b) The HPLC chromatogram of ethyl acetate fraction of *E. koreanum* (EAF) was detected at 280 nm UV.

To analyze the phenolic compound contents in the *E. koreanum* ethyl acetate fraction (EAF), we compared it with standard substances (gallic acid, chlorogenic acid, caffeic acid, ellagic acid, myricetin, icariin, quercetin, and kaempferol) (Figure 6(a)). Based on the HPLC analysis, icariin was detected in the ethyl acetate fraction (tR = 17.59 min) and its contents were 58.75 mg/g (Figure 6(b)).

## 4. Discussion

Platelets play a major role in maintaining hemostasis and healing injured vessels to stop blood loss. However, aberrant platelet activation leads to thrombus formation within blood vessels, which may cause thromboembolism, eventually leading to stroke, heart attack, and deep vein thrombosis, among other morbidities. Available common anti-platelet drugs possess adverse effects, and some patients may be resistant to them [20], which necessitates the development of alternate approaches that could be best in the form of natural products. Many natural compounds have been reported to possess anti-platelet and anti-thrombotic properties with minimum or no side effects, and have also been useful in CVD treatment and prevention [21, 22]. *E. koreanum* Nakai has been reported for its anti-oxidant, anti-aging, and anti-atherosclerotic, as well as sexual boosting properties, and is also known for its cardio-protective effects [12–14].

In the present study, we evaluated its anti-platelet properties. Our initial screening results indicated that *E. koreanum* inhibited platelet aggregation against several agonists, but more potently against collagen, in both human and rat platelets. The possible reason to this could be attributed to the active ingredients contained in the ethyl acetate fraction, which may require further study and evaluation. We confirmed the platelet aggregation and shape changes by observing the ultrastructure using SEM, which clearly showed dose-dependent inhibition of aggregation in platelets pre-treated with the fraction. Agonist-stimulated elevation in cytosolic Ca^2+^ concentrations is essential for platelet activation in hemostasis and thrombosis [23], whereas dense granule secretion (ATP, ADP, and Ca^2+^) further enhances platelet adhesion, shape change, and aggregation [21]. Our results indicated that *E. koreanum* fraction potently inhibited [Ca^2+^]_*i*_ mobilization and ATP secretion, thereby inhibiting platelet activation.

Conformational changes in integrin *α*_IIb_*β*_3_ structure caused an enhanced ability to bind to fibrinogen (inside-out signaling), whereas this phenomenon further transduces signals into the cell (outside-in signaling), leading to platelet adhesion, spreading, and clot retraction [24]. Similarly, fibronectin is another adhesive ligand that stabilizes thrombus formation in blood vessels. It binds to integrins and augments platelet aggregation by developing cohesive aggregates [25]. Rho kinases (ROCKs) are downstream regulators of GTPases, which mediate RhoA-stimulated actin cytoskeletal changes via myosin light chain phosphorylation. The role of ROCKs in facilitating clot retraction has been previously described using ROCK inhibitor (Y-27632) [26]. Moreover, Src kinase and PLC*γ*2 involvement in clot retraction has been reported [27]. *E. koreanum* significantly inhibited fibrinogen-integrin *α*_IIb_*β*_3_ binding, fibronectin adhesion, and clot retraction, indicating its potential to modulate integrin-mediated inside-out and outside-in signaling. The fraction also dose-dependently inhibited Src and PLC*γ*2 phosphorylation, which further confirms their involvement in inhibiting platelet functions and clot retraction.

**Figure 7:**
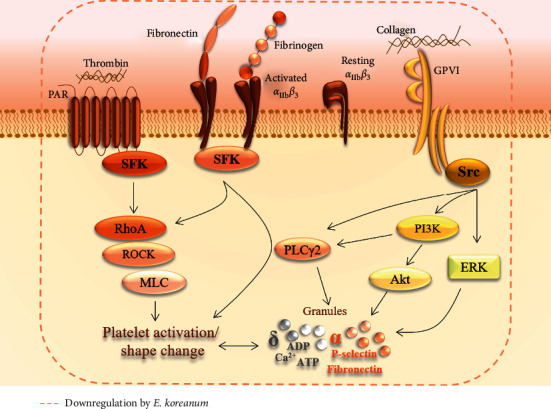
A schematic summary of the inhibitory effects of *E. koreanum* on intracellular platelet signaling pathway.

Src is involved in early signaling of platelets and plays a key role in various downstream signaling pathways [28]. MAPK (ERK and P38^MAPK^) causes granule secretion and platelet aggregation [29]. Similarly, Akt has been reported to regulate platelet activation with potential consequences, such as thrombosis, and is a very important target in designing anti-thrombotic drugs [30]. The fraction's underlying inhibitory mechanisms on platelet functions were further examined by assessing the aforementioned pathways, and we found that *E. koreanum* inhibited Src, ERK, Akt, and PLC*γ*2 phosphorylation.  Figure 7 summarizes the inhibitory effects of *E. koreanum* on intracellular platelet signaling pathways.

The AV-shunt model is commonly used to assess *in vivo* antithrombotic effects. The shunt was previously recognized to be made up of platelet, fibrin, and red blood cells trapped inside, and such thrombus may lead to coronary artery disease or myocardial infarction; however, this phenomenon could be reversed by antiplatelet agents [31]. In our results, *E. koreanum* fraction potently attenuated the thrombus formation and moderately increased bleeding time in mice, which indicated that the fraction inhibited platelet activation and aggregation.

HPLC is very helpful in identifying and characterizing the chemical profiles of natural products [32]. Our results revealed that the fraction predominantly contains icariin, a drug that has been reported for several pharmacologic functions, including antiatherosclerotic properties [33] and attenuation of the prothrombotic state of atherosclerosis [34]. The results suggest that the observed inhibitory effects of the fraction treatment on platelets could be attributed to icariin that is mainly present in *E. koreanum*.

## 5. Conclusion

The ethyl acetate fraction of *E. koreanum* (EAF) inhibited platelet aggregation in rat and human platelets, attenuated (Ca^2+^)_*i*_ mobilization and ATP secretion, and modulated integrin-mediated inside-out and outside-in signaling via Src, ERK, and Akt inhibition. The fraction has also inhibited thrombus formation, indicating its possible therapeutic effects, which suggest that *E. koreanum* is a potential candidate to treat and prevent platelet-related cardiovascular disorders in the new era of ethnomedicine.

## Data Availability

The datasets used and/or analyzed during the current study are available from the corresponding author upon reasonable request.

## Abbreviations

ACD: Acid-citrate-dextrose
ADP: Adenosine diphosphate
ASA: Acetylsalicylic acid; aspirin
AV shunt: Arteriovenous shunt
Akt: Protein kinase B
DMSO: Dimethyl sulfoxide
EAF: Ethyl acetate fraction of *Epimedium koreanum*
ERK: Extracellular signal-regulated kinase
GPVI: Glycoprotein VI
MAPK: Mitogen-activated protein kinase
Rho-A: Rho kinase A
SEM: Scanning electron microscope
SFK: Src family kinase
TXB2: Thromboxane-B2.

## Supplementary Materials

Supplementary Materials Detailed description on chemicals and reagent sources, animals and handling, preparation of washed human and rat platelets, scanning electron microscopy (SEM), ATP secretion and TXB2 production, measurement of intracellular calcium mobilization, flow cytometry, fibronectin adhesion assay, clot retraction, immunoblotting, *in vivo* AV-shunt assay, and bleeding time has been included in the Supplementary Materials. We followed the methods of Irfan et al. [17].
