# Supplementary material for: Antiplatelet and Antithrombotic Effects of Epimedium koreanum Nakai
Source: Evid Based Complement Alternat Med. 2021 Apr 16;2021:7071987. doi: 10.1155/2021/7071987 (PMC8068545; doi:10.1155/2021/7071987)
Supplement: Supplementary Materials — Detailed description on chemicals and reagent sources, animals and handling, preparation of washed human and rat platelets, scanning electron microscopy (SEM), ATP secretion and TXB2 production, measurement of intracellular calcium mobilization, flow cytometry, fibronectin adhesion assay, clot retraction, immunoblotting, in vivo AV-shunt assay, and bleeding time has been included in the Supplementary Materials. We followed the methods of Irfan et al. [17]. [file 7071987.f1.docx]

**Supplementary Material**

**Anti-platelet and anti-thrombotic effects of *Epimedium koreanum* Nakai**

Muhammad Irfan^1,2,#^, Tae-Hyung Kwon^3,#^, Dong-Ha Lee^4^, Seung-Bok Hong^5^, Jae-Wook Oh^6^, Sung-Dae Kim^1,7**^ and Man Hee Rhee^1,*^

^1^ Department of Veterinary Medicine, College of Veterinary Medicine, Kyungpook National University, Daegu 41566, Republic of Korea

^2^ Department of Oral Biology, College of Dentistry, University of Illinois at Chicago, Chicago 60607, IL, USA.

^3^ Chuncheon Bio Industry Foundation, Chuncheon 24232, Republic of Korea.

^4^ Department of Clinical Laboratory Science, Chungbuk Health & Science University, Chungbuk 28150, Republic of Korea

^5^ Department of Biomedical Laboratory Science; and Molecular Diagnostics Research Institute, Namseoul University, Cheonan 31020, Republic of Korea

^6^ Department of Stem Cell and Regenerative Biotechnology, KIT, Konkuk University, Seoul, 05029 Republic of Korea

^7^ Research Center, Dongnam Institute of Radiological and Medical Sciences, Busan 46033, Republic of Korea

***Correspondence:**

Man Hee Rhee, PhD

Laboratory of Physiology and Cell Signaling, College of Veterinary Medicine, Kyungpook National University, Daegu 41566, Republic of Korea

Tel: +82-53-950-5967; Fax +82-53-950-5955

*e*-mail: rheemh@knu.ac.kr

***2. Material and Methods***

***2.1. Chemicals and reagents***

Collagen (native collagen fibrils from equine tendons; type I), adenosine diphosphate (ADP), and thrombin were acquired from Chrono-log (Havertown, PA, USA). Fura-2-acetoxymethyl ester (fura-2/AM), dimethyl sulfoxide (DMSO), ASA, and all standard compounds (*i.e.*, gallic acid, chlorogenic acid, caffeic acid, ellagic acid, myricetin, icariin, quercetin, kaempferol) used for analysis of phenolic compounds were purchased from Sigma-Aldrich (St. Louis, MO, USA). The ATP Assay kit was obtained from Biomedical Research Service Center (Buffalo, NY, USA), whereas Fibrinogen Alexa Fluor 488 conjugate and Fibronectin adhesion assay kit were procured from Molecular Probes (Eugene, OR, USA) and Cell Biolabs Inc. (San Diego, CA, USA), respectively. Antibodies against extracellular signal-regulated kinases (ERK; p44/42), phospho-ERK (phospho-p44/42), p38^MAPK^, phospho-p38^MAPK^, Akt, phospho-Akt, phospho-Src, phspho-PLCγ2, and β-actin were acquired from Cell Signaling Technology (Beverly, MA, USA). Water was obtained from J. T. Baker (Phillipsburg, NJ, USA). All chemicals were of reagent grade.

***2.2. Animals***

Seven-week-old male Sprague–Dawley rats weighing 220–240 g and 7-week-old C57BL/6J male mice weighing 20–22 g, were purchased from Orient Co. (Seoul, Republic of Korea) and acclimatized for 1 week in an animal room maintained at 23 °C ± 2 °C and 50% ± 10% humidity under a 12-h light/dark cycle before the actual experiment. The experiments were conducted according to IACUC guidelines, and experimental protocols were approved by the Ethics Committee of the College of Veterinary Medicine, Kyungpook National University, Daegu, Korea (KNU12-125). Later, rats were euthanized by an overdose of 5% isoflurane as previously described [1]. Isoflurane exposure was continued after 1 min of breathing stoppage, followed by cervical dislocation for confirmation of euthanasia.

***2.3. Preparation of washed human and rat platelets***

Human platelet-rich plasma (PRP) collected from healthy volunteers, who provided informed consent, was obtained from the Korean Red Cross Blood Center (KRBC, Changwon, Korea), and its experimental use was approved by KRBC and the Korea National Institute for Bioethics Policy Public Institutional Review Board (PIRB17-1019-03). The washed human platelets were prepared and adjusted to a final concentration of 5 × 10^8^/mL as previously described [2].

To prepare rat washed platelets, whole blood was collected from rats via heart puncture, then anti-coagulated with ACD solution. Blood was centrifuged at 170 ×g for 7 min to obtain PRP. Subsequently, PRP was centrifuged at 350 ×*g* for 7 min to isolate the washed platelets. Platelet concentration was adjusted to 3 × 10^8^ cells/mL using Tyrode’s buffer (137 mM NaCl, 12 mM NaHCO_3_, 5.5 mM glucose, 2 mM KCl, 1 mM MgCl_2_, and 0.3 mM NaHPO_4_; pH 7.4) for platelet aggregation assays. All preparatory procedures were performed at room temperature (23 °C ± 2 °C). We followed the methods of Irfan et al., [3]

***2.4. Scanning electron microscope (SEM) analyses***

A field emission SEM was used to assess platelet shape change and aggregation by obtaining ultrastructure images. After the platelet aggregation assay, the platelet mixture was treated with 0.5% paraformaldehyde (first fixation) for 30 min at 4ºC and then with osmium tetroxide (second fixation) for 4 hours at 4ºC, dehydrated with ascending concentrations of ethanol (50 to 100%), freeze-dried in a Lyophilizer, and subsequently scanned under scanning electron microscope as previously described [3, 4].

***2.5. Assessment of ATP release and thromboxane-B2 production***

Washed rat platelets (3 × 10^8^ cells/mL) were incubated with different dosages of either EAF or vehicle for 1 min in the presence of 1 mM CaCl_2_ and then stimulated with agonist (collagen) for 5 min at 37 °C with continuous stirring as previously described method [5]. Aggregation reaction was terminated by keeping the platelet suspension on the ice, and the supernatant was obtained by centrifuging the platelet mixture, then ATP secretion was measured on a luminometer (GloMax 20/20; Promega, Madison, WI, USA) using an ATP assay kit (Biomedical Research Service Center) and thromboxane-B2 production was assessed using TXB2 ELISA kit (Enzo Life Sciences, USA).

***2.6. Measurement of [Ca^2+^]_i_ mobilization***

Intracellular calcium ion ([Ca^2+^]*_i_*) concentration was assessed with Fura-2/AM as previously described method [6]. PRP was incubated with 5 µM Fura-2/AM for 1 h at 37 °C. Next, Fura-2/AM-loaded washed platelets (3 × 10^8^ cells/mL) were pre-incubated with either EAF or vehicle for 1 min in the presence of 1 mM CaCl_2_ and stimulated with collagen for 3 min. Fura-2 fluorescence in the cytosol was measured using a spectrofluorometer (F-2500; Hitachi) and calculated using the following formula, as described by Schaeffer and Blaustein [7]: [Ca^2+^]*_i_* = 224 nM × (*F* − *F_min_*)/(*F_max_* − *F*); where, 224 nM is the dissociation constant of the Fura-2-Ca^2+^complex, and *F_min_* and *F_max_* are the fluorescence intensities at very low and very high Ca^2+^ concentrations, respectively.

***2.7. Flow cytometry***

Effects of EAF on fibrinogen binding was measured via flow-cytometry using Alexa Fluor 488‐human fibrinogen as previously reported method [8]. Briefly, the pre-treated washed platelets (3 × 10^8^ cells/mL) with vehicle or different dosages of EAF were stimulated with collagen for 5 min. Following, treatment with Alexa Fluor 488‐human fibrinogen (20 µg/mL) for 5 min at room temperature and fixation with 0.5% paraformaldehyde for 30 min at 4°C. Suspension was further washed with Phosphate buffered saline and reconstituted for further analysis. Flow cytometric analysis was performed using a FACS Aria™ III flow cytometer® (Becton Dickinson Immunocytometry Systems, San Jose, CA, USA), and the data were analyzed using the CellQuest software (Becton Dickinson Immunocytometry Systems).

***2.8. Fibronectin adhesion assay***

To determine the effects of EAF on platelet adhesion, a fibronectin adhesion assay was performed as previously reported method [3], using the Fibronectin Adhesion Assay kit (Cell Biolabs, Inc.), according to the manufacturer’s instructions. Briefly, the washed platelets (1 × 10^8^ cells/mL) pre-treated with vehicle, different concentrations of EAF, or GR155053 (α_IIb_β_3_ inhibitor; as positive control) were incubated in fibronectin-coated wells for 90 min at 37°C and then washed with double-distilled water (DDW). Following, staining of the adhered cells with crystal violet for 10 min and washed with DDW. Finally, the stained adhered cells were solubilized with extraction solution (Acetic acid solution). The obtained lysate solution was transferred to a 96-well plate, and absorbance was measured at 540 nm.

***2.9. Clot retraction and kinetics of clot retraction***

PRP (250 µL; 5 × 10^8^ cells/mL) was mixed with red blood cells (5 µL) and incubated for 2 min with vehicle, or different concentrations of EAF, or Y-27632 (ROCK inhibitor). Then, tyrode’s buffer was added to a final volume of 1 mL as previously reported method [9]. Thrombin (1 U/mL) was added to trigger fibrin clot formation, and observed for 2 h at room temperature and photographed at 15-min intervals. Clot weight was measured as a marker of clot retraction. The kinetics of clot retraction were assessed as previously described method [10]. The pictures were processed using the ImageJ software, and clot surface areas were plotted as a percentage of clot retraction.

***2.10. Immunoblotting***

Washed platelets (3 × 10^8^ cells/mL) were pre-incubated with various concentrations of *E. koreanum,* along with 1 mM CaCl_2_ for 1 min at 37 °C, and then stimulated with collagen for 5 min under continuous stirring. Platelet aggregation was terminated by adding a lysis buffer (PRO-PREP; iNtRON Biotechnology, Seoul, Korea), and protein concentration was estimated using BCS assay (PRO-MEASURE; iNtRON Biotechnology). The total platelet proteins were separated in 10% SDS–PAGE and transferred to polyvinylidene fluoride (PVDF) membranes. Membranes were blocked with 5% skimmed milk, probed with respective antibodies (*i.e.*, ERK, phospho-ERK, p38^MAPK^, phospho-p38^MAPK^, Akt, phospho-Akt, phospho-Src, phspho-PLCγ2, and β-actin), and visualized using enhanced chemiluminescence and phosphorylation ratio was calculated using ImageJ software. We followed the methods of Irfan et al., [3].

***2.11. Arteriovenous shunt model***

The anti-thrombotic activity of the EAF was assessed in a rat extracorporeal shunt model as previously described methods [3, 11]. Briefly, SD rats were orally administered with the saline (control), EAF (100-300 mg/kg), or ASA (50 mg/kg) once daily for 3 days. Two hours after the last administration, rats were anesthetized with urethane (1.75 g/kg i.p.) and an incision was made over the trachea. The right jugular vein and left carotid artery were exposed, and the two ends of the extracorporeal shunt were inserted into them. The shunt consisted of two 12 cm long polyethylene tube (0.81 mm and 0.58 mm external and internal diameter, respectively), which is connected to a 6 cm long polyvinyl tube (3-mm internal diameter) by 5-mm silicone rubber plugs. A 6 cm long cotton thread was secured between the two plugs so that it remains longitudinally orientated in the blood flowing through the cannula. Before cannulation, the tube was filled with 0.9% saline solution. The shunt was left in place for 15 min after initiating extracorporeal circulation. Subsequently, blood flow was stopped, thread was removed, and the thrombus formed was separated from the thread and unclotted blood and weighed.

***2.12. In vivo bleeding assay***

Male C57BL/6J mice were divided into three treatment groups (n = 5), and intraperitoneally administered with saline (control), ASA (50 mg/kg), or EAF (300 mg/kg) once daily for 3 days. One hour after the last administration, mice were anesthetized, and tail bleeding assay was performed as previously described [8, 12]. Briefly, the tail was pre-warmed for 3 min in normal saline solution at 37 °C and bleeding was induced by a precise incision on the mouse tail, 3-5 mm from the tip. The distal portion of the tail (3 cm) was vertically immersed into 0.9% saline solution at 37 °C. The time from transection initiation to bleeding cessation was recorded as the bleeding time.

**References**

1. Jeong D, Irfan M, Lee D-H, Hong S-B, Oh J-W, Rhee MH: Rumex acetosa modulates platelet function and inhibits thrombus formation in rats. *BMC complementary medicine and therapies* 2020, 20(1):1-9.

2. Irfan M, Jeong D, Kwon H-W, Shin J-H, Park S-J, Kwak D, Kim T-H, Lee D-H, Park H-J, Rhee MH: Ginsenoside-Rp3 inhibits platelet activation and thrombus formation by regulating MAPK and cyclic nucleotide signaling. *Vascular pharmacology* 2018, 109:45-55.

3. Irfan M, Kwon H-W, Lee D-H, Shin J-H, Yuk HJ, Kim D-S, Hong S-B, Kim S-D, Rhee MH: Ulmus parvifolia Modulates Platelet Functions and Inhibits Thrombus Formation by Regulating Integrin αIIbβ3 and cAMP Signaling. *Frontiers in Pharmacology* 2020, 11:698.

4. White JG: Electron microscopy methods for studying platelet structure and function. In: *Platelets and Megakaryocytes.* edn.: Springer; 2004: 47-63.

5. Kim DS, Irfan M, Sung YY, Kim SH, Park SH, Choi YH, Rhee MH, Kim HK: Schisandra chinensis and Morus alba Synergistically Inhibit In Vivo Thrombus Formation and Platelet Aggregation by Impairing the Glycoprotein VI Pathway. *Evidence-based complementary and alternative medicine : eCAM* 2017, 2017:7839658.

6. Jeong D, Irfan M, Kim S-D, Kim S, Oh J-H, Park C-K, Kim H-K, Rhee MH: Ginsenoside Rg3-enriched red ginseng extract inhibits platelet activation and in vivo thrombus formation. *Journal of ginseng research* 2017, 41(4):548-555.

7. Schaeffer J, Blaustein MP: Platelet free calcium concentrations measured with fura-2 are influenced by the transmembrane sodium gradient. *Cell Calcium* 1989, 10(2):101-113.

8. Endale M, Lee WM, Kamruzzaman SM, Kim SD, Park JY, Park MH, Park TY, Park HJ, Cho JY, Rhee MH: Ginsenoside-Rp1 inhibits platelet activation and thrombus formation via impaired glycoprotein VI signalling pathway, tyrosine phosphorylation and MAPK activation. *British journal of pharmacology* 2012, 167(1):109-127.

9. Tucker KL, Sage T, Gibbins JM: Clot retraction. In: *Platelets and Megakaryocytes.* edn.: Springer; 2012: 101-107.

10. Misztal T, Przesław K, Rusak T, Tomasiak M: Peroxynitrite–altered platelet mitochondria—A new link between inflammation and hemostasis. *Thrombosis research* 2013, 131(1):e17-e25.

11. Irfan M, Kwon T-H, Yun B-S, Park N-H, Rhee MH: Eisenia bicyclis (brown alga) modulates platelet function and inhibits thrombus formation via impaired P2Y12 receptor signaling pathway. *Phytomedicine* 2018, 40:79-87.

12. Irfan M, Jeong D, Saba E, Kwon H-W, Shin J-H, Jeon B-R, Kim S, Kim S-D, Lee D-H, Nah S-Y: Gintonin modulates platelet function and inhibits thrombus formation via impaired glycoprotein VI signaling. *Platelets* 2019, 30(5):589-598.
